# Delineation and mapping of coastal shark habitat within a shallow lagoonal estuary

**DOI:** 10.1371/journal.pone.0195221

**Published:** 2018-04-12

**Authors:** Charles W. Bangley, Lee Paramore, Simon Dedman, Roger A. Rulifson

**Affiliations:** 1 Institute for Coastal Science and Policy, East Carolina University, Greenville, NC, United States of America; 2 Smithsonian Environmental Research Center, Edgewater, MD, United States of America; 3 North Carolina Division of Marine Fisheries, Northern District Office, Elizabeth City, North Carolina, United States of America; 4 Farallon Institute, Petaluma, CA, United States of America; 5 Department of Biology, East Carolina University, Greenville, NC, United States of America; Griffith University, AUSTRALIA

## Abstract

Estuaries function as important nursery and foraging habitats for many coastal species, including highly migratory sharks. Pamlico Sound, North Carolina, is one of the largest estuaries in the continental United States and provides a variety of potential habitats for sharks. In order to identify and spatially delineate shark habitats within Pamlico Sound, shark catch and environmental data were analyzed from the 2007–2014 North Carolina Division of Marine Fisheries (NCDMF) gillnet and longline surveys conducted within the estuary. Principal species were identified and environmental data recorded at survey sites (depth, temperature, salinity, dissolved oxygen, submerged aquatic vegetation (SAV) distance, and inlet distance) were interpolated across Pamlico Sound to create seasonal environmental grids with a 90-m^2^ cell size. Boosted Regression Tree (BRT) analysis was used to identify the most important environmental factors and ranges associated with presence of each principal species, and the resulting models were used to predict shark capture probability based on the environmental values within the grid cells. The Atlantic Sharpnose Shark (*Rhizoprionodon terraenovae*), Blacktip Shark (*Carcharhinus limbatus*), Bull Shark (*Carcharhinus leucas*), Sandbar Shark (*Carcharhinus plumbeus*), Smooth Dogfish (*Mustelus canis*), and Spiny Dogfish (*Squalus acanthias*) were the principal species in Pamlico Sound. Most species were associated with proximity to the inlet and/or high salinity, and warm temperatures, but the Bull Shark preferred greater inlet distances and the Spiny Dogfish preferred lower temperatures than the other species. Extensive Smooth Dogfish habitat overlap with seagrass beds suggests that seagrass may be a critical part of nursery habitat for this species. Spatial delineation of shark habitat within the estuary will allow for better protection of essential habitat and assessment of potential interactions with other species.

## Introduction

Estuaries often function as important habitats for marine species, though the dynamic nature of the transition from freshwater to saltwater has a significant effect on habitat use and availability [1]. Within these environments, habitat selection and use patterns are influenced by abiotic factors such as temperature and salinity [2] and biotic factors such as food availability and predation risk [3,4]. Often abiotic and biotic factors interact, such as when a species selects habitat outside the preferred environmental ranges of its predators [5,6] or competitors [7].

Though often associated with juvenile teleosts and invertebrates, estuaries also function as nursery and foraging habitats for coastal sharks [8]. Like other species, sharks select estuarine habitats based on a combination of abiotic environmental tolerances and biotic factors such as prey availability, predator avoidance, and competition [9]. Most studies on shark habitat use within estuaries have focused on juveniles or small-bodied species, but the ecological role of sharks can change dramatically with increasing size [10]. Juvenile and small coastal sharks appear to select habitat that minimizes predation risk or competition [11,12] though this can come at the expense of foraging opportunities [13,14]. However, other species occur in habitats with far greater predation risk, seeming to prioritize feeding over protection [15]. Large, apex predatory sharks can exert direct and indirect top-down effects that may have observable landscape effects on estuarine habitats [16,17] and influence the habitat use patterns of other predators, including other elasmobranchs [18,19]. Even juvenile or small-bodied sharks can occupy similar trophic niches to high-trophic level teleosts and other estuarine predators, possibly functioning as competitors or predators of species of value to fisheries [20,21].

Identification of important shark habitat is complicated by their generally highly mobile nature [22]. However, recurrent site fidelity to specific areas or habitats is widespread even among highly migratory shark species [23,24]. Fidelity to nearshore or estuarine habitats can concentrate otherwise wide-ranging sharks into areas with an increased exposure to anthropogenic affects such as fishing pressure, pollution, and coastal development [24,25]. Seasonal or regular presence of sharks in an estuary can also cause changes in the behavior and distribution of other species within the system with possible cascading ecological effects [26]. Therefore, identifying and delineating estuarine shark habitat is important to both managing shark populations and broader ecosystem-based management.

Pamlico Sound, North Carolina’s largest estuary and the largest barrier island lagoon system in the United States, potentially includes a wide variety of shark habitats. Pamlico Sound has the greatest proportion of open water of any U.S. barrier island estuary, but is relatively shallow in comparison to other large estuaries [27]. High salinity water enters the system from the southeast through Hatteras Inlet, Ocracoke Inlet, and Core Sound and exits in the northern extent of the estuary through Oregon Inlet [28]. Fresh water flows into the estuary from the west through the Neuse and Pamlico Rivers, and also enters from Albemarle Sound north of Oregon Inlet. This creates a horizontal salinity ecocline with salinity increasing west to east [28]. Pamlico Sound is situated at a biogeographic break, delineated at Cape Hatteras, between temperate and subtropical marine ecosystems [29,30]. Due to its biogeographic position, Pamlico Sound encompasses the majority of the range overlap between the temperate seagrass *Zostera marina* and the subtropical seagrass *Halodule wrightii*, which combine to give the estuary one of the greatest areas of seagrass habitat on the U.S. East Coast [31,32]. Because of its high diversity of habitats, Pamlico Sound is uniquely situated to support a diverse shark community that varies both spatially and seasonally.

Though biotic factors such as prey availability and competitive exclusion can be important in determining the distribution and habitat use of sharks in an estuary, delineating habitat based on spatial and abiotic environmental factors is likely more tractable and more easily applied to management applications [33]. Here we use fishery-independent survey data to describe the coastal shark community within Pamlico Sound, determine the environmental preferences of the principal species in this community, and spatially delineate habitat for these sharks.

## Methods

### Field data collection and processing

**Fig 1:**
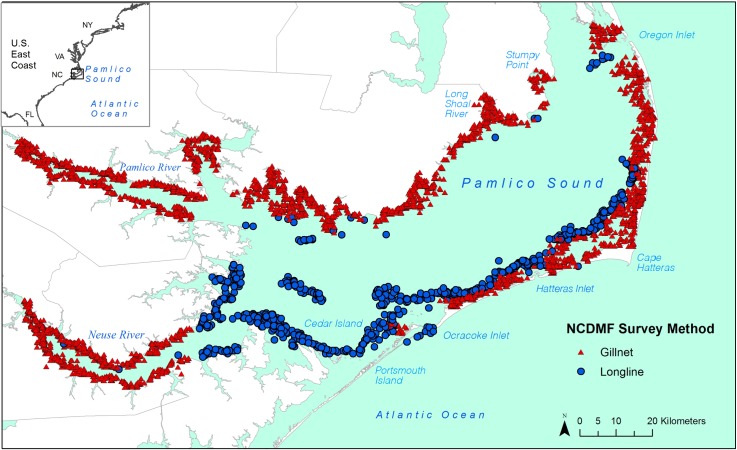
North Carolina Division of Marine Fisheries gillnet and longline survey stations within Pamlico Sound from 2007–2014. Gillnet sets are marked with red triangles and longline sets are marked with blue circles.

Shark catch and environmental data were obtained from the NCDMF fishery-independent gillnet and longline surveys conducted within Pamlico Sound. The study period ranged from 2007 through 2014 for both surveys. The gillnet survey has been conducted since 2001, but only 2007–2014 data were used to match temporal coverage with the longline survey. The total combined area of the surveys covered the east side of the sound from Oregon Inlet to the entrance of Core Sound near Portsmouth Island, and the west side of the sound from Stumpy Point to the entrance of Core Sound near Cedar Island including the estuarine portions of the Neuse and Pamlico Rivers (Fig 1). Gillnet and longline set locations were selected using a stratified-random sampling design in which the total study area from the shoreline to approximately the 2-m depth contour was divided into eight substrata, then further divided into 1.85 by 1.85-km cells that were chosen at random before each month of sampling. Effort was composed of 619–624 sets per year for the gillnet survey and 72–137 sets per year for the longline survey, though starting in 2011 longline effort was standardized at 72 sets per year. Gillnet sampling was conducted from February through December of each year, while longline sampling was conducted from July through November. Gillnet gear was composed of a 219.46-m sink gillnet made up of eight 27.43-m sections of 7.62, 8.89, 10.16, 11.43, 12.70, 13.97, 15.24, and 16.51-cm stretched width mesh soaked for 12 hours from sunset to sunrise. Longline gear consisted of a 1500-m mainline with 100 gangions attached at 15-m intervals, each of which was made up of 0.7 m of 91-kg test monofilament and a 15/0 circle hook baited with Striped Mullet (*Mugil cephalus*) or locally available bait fish, which was allowed to soak for 30 minutes per set. All survey procedures followed standard NCDMF protocols and were authorized by the North Carolina state statute Article 20 § 113–261.

All captured sharks were identified to species and total length (TL, mm) and sex were recorded. At each station, depth (m), water temperature (°C), salinity (ppt), and dissolved oxygen concentration (mg/L) was recorded prior to setting gear. After sampling, straight-line distances from the nearest inlet (km) and nearest seagrass bed (m) were measured for each set location using the spatial join function in ArcGIS 10.1 (ESRI). Inlet distance was measured to the nearest opening connecting Pamlico Sound to the Atlantic Ocean. Locations and spatial extents of seagrass beds were taken from maps generated using data collected from aerial and boat-based surveys conducted from 2006 through 2010 by the Albemarle-Pamlico National Estuarine Partnership (APNEP) [34].

All species with more than 40 individuals captured during the survey period were designated as principal species for further analysis. The number of sharks captured in both gears combined was plotted by year, month, and season. Six seasons were designated by calendar date: December 1-February 29 was classified as Winter, March 1-April 15 as Early Spring, April 16-May 31 as Late Spring, June 1-August 30 as Summer, September 1-October 15 as Early Fall, and October 16-November 31 as Late Fall. For each species, the season in which that species was captured in the greatest numbers was used for habitat delineation. In order to determine the possibility of habitat overlap, linear discriminant function analysis (DFA) was used to classify the principal species using the six environmental factors (depth, temperature, salinity, dissolved oxygen, seagrass distance, and inlet distance). Species either in close proximity on the canonical plot or frequently misclassified as each other were considered to have overlapping habitat preferences.

Though the gillnet made use of multiple mesh sizes to decrease size selectivity bias, considerable differences in shark size and species selectivity can occur between gear types [35,36]. In addition, the differences in soak time and sampling season between the longline and gillnet surveys likely violated assumptions of equal catchability and precluded the use of catch-per-unit-effort (CPUE) to measure species abundance. Because of this, likelihood of species presence was used to model habitat, and was represented as the probability of capture in both gears combined. Likelihood of species presence was assumed to be equal between the two gear types, but this could not be conclusively assessed due to a lack of spatial and temporal overlap between surveys.

### Habitat delineation

Habitat delineation followed the approach used by Froeschke et al. [37] and Dedman et al. [38], in which boosted regression tree analysis was used to predict capture probability based on environmental factors, which was then mapped. BRT analysis was used because it provides a number of advantages in modeling habitat in situations such as highly zero-inflated and long-tailed catch data common in elasmobranch surveys, while reducing the variance that may bias habitat delineation using single regression trees [38,39]. Habitat delineation proceeded in two stages: first, relationships between environmental factors and species capture probability were identified using BRTs, then these relationships were used to predict potential abundance throughout the entire area of Pamlico Sound covered by NCDMF surveys [38]. To accomplish this, the function *gbm*.*auto* was used to automate several functions of the *dismo* and *gbm* packages in R [40,41].

### Environmental data processing

Prior to BRT analysis, temperature, salinity, dissolved oxygen, SAV distance, and inlet distance measurements at NCDMF gillnet and longline stations were interpolated across Pamlico Sound using Bayesian Empirical Kriging in ArcGIS 10.1. Temperature, salinity, and dissolved oxygen were expected to vary by season so these environmental layers were interpolated for each season. Interpolated layers were then converted to raster grids with a 90-m^2^ cell size. Depth was represented by a raster of bathymetry data collected by the U.S. Geological Survey (USGS). Negative values were changed to zeroes in the grid files to ensure models represented a natural range of environmental measurements. Data from environmental rasters were extracted and combined into seasonal .csv files for import into R during BRT modeling. At this stage, multiple linear correlation analysis was used to identify any strong autocorrelations between environmental factors, though BRT analysis is robust to autocorrelation.

### BRT modeling

In R, *gbm*.*auto* used machine learning to automatically split the probability space explaining study species presence as predicated on environmental variables at key breakpoints along those variables’ ranges. This generated sets of regression trees describing increasingly nuanced relationships between shark presence/absence and environmental variable values [38,39]. Binary presence/absence trees used data from all sets to model capture probability. In addition, the function generated marginal effect plots for each environmental factor showing the positive or negative effect of that factor on capture probability of each species, as well as the contribution of that factor to the overall model based on the percentage of tree splits associated with it. Models were run with different combinations of learning rate (lr) and bag fraction (bf) until the model with the highest cross-validation (CV) score producing consistent results was identified [39]. Model results were considered consistent if the order of the importance of habitat factors was the same and the percentage of tree splits associated with each factor did not differ by more than 5% over three consecutive runs.

After BRT analysis, the predictor model was applied to the environmental raster grid data from the season in which the species was most abundant, which created predicted capture probability values based on the environmental factors in each grid cell. These data were then imported back into ArcGIS and interpolated using Bayesian Empirical Kriging to depict a predicted capture probability surface for each principal species within Pamlico Sound. Maps of unrepresentativeness, which measured the extent to which the full range of environmental conditions were represented [40], were also plotted for each season.

Habitat models were validated by overlaying catch data for each species from 2015 NCDMF gillnet and longline surveys over their respective habitat map and extracting the predicted capture probability at each survey set location. The 2015 survey year was used because it immediately followed the time series of data used to generate the predicted habitat maps, increasing the likelihood that the spatial distribution of shark capture locations would not be influenced by long-term environmental trends. For each species, survey stations were classified as either present or absent and a Kruskal-Wallis test was used to determine whether capture probability was greater in sets in which the species was present.

## Results

### Field data collection and processing

A total of 2,408 sharks were captured from 2007–2014 in NCDMF surveys within Pamlico Sound. In all, 11 species were represented with an additional 44 individuals classified as either unknown or misidentified Carcharhinidae (Table 1). Misidentified sharks were designated based on mismatches between the measured TL and published size ranges for the recorded species name. Individuals within neonatal size ranges were present among Atlantic Sharpnose Sharks (*Rhizoprionodon terraenovae*), Bull Sharks (*Carcharhinus leucas*), Finetooth Sharks (*Carcharhinus isodon*), Sandbar Sharks (*Carcharhinus plumbeus*), Smooth Dogfish (*Mustelus canis*), and Spinner Sharks (*Carcharhinus brevipinna*). The Atlantic Sharpnose Shark, Blacktip Shark (*Carcharhinus limbatus*), Bull Shark, Sandbar Shark, Smooth Dogfish, and Spiny Dogfish (*Squalus acanthias*) were designated as the principal species for further analysis (Table 1).

**Table 1:** Total number captured (number of sets occurring for principal species in parentheses) and mean ± standard deviation total length (TL) and environmental factor measurements (minimum and maximum in parentheses) for each shark species captured during North Carolina Division of Marine Fisheries gillnet and longline surveys within Pamlico Sound. Species denoted with an asterisk were designated principal species.

Species	Total n (n sets)	TL (mm) (Min-Max)	Depth (m) (Min-Max)	Temp (°C) (Min-Max)	Sal (ppt) (Min-Max)	DO (mg/L) (Min-Max)	SAV Dist (m) (Min-Max)	Inlet Dist (km) (Min-Max)
*Atlantic Sharpnose Shark *Rhizoprionodon terraenovae*	98 (44)	722.9 ± 209.1 (338–1040)	1.7 ± 0.7 (0.6–4.0)	26.1 ± 2.6 (18.3–30.6)	28.2 ± 5.0 (17.1–35.6)	7.1 ± 0.9 (5.3–11.6)	639.6 ± 732.9 (0.0–2773.5)	8.3 ± 5.6 (0.2–39.7)
*Blacktip Shark *Carcharhinus limbatus*	52 (26)	1305.7 ± 108.5 (819–1638)	2.1 ± 0.7 (0.4–3.4)	28.6 ± 1.6 (24.4–32.0)	24.9 ± 5.0 (12.4–35.6)	6.9 ± 1.1 (3.5–10.4)	1038.5 ± 1138.9 (0.0–5373.2)	7.8 ± 3.8 (0.0–21.7)
Bonnethead Shark *Sphyrna tiburo*	21	909.7 ± 86.1 (748–1104)	1.6 ± 0.6 (0.6–2.3)	28.3 ± 1.8 (25.6–31.4)	26.7 ± 3.5 (19.8–31.2)	7.6 ± 1.3 (5.6–9.3)	40.3 ± 54.2 (0.0–203.9)	13.7 ± 10.0 (3.6–32.0)
*Bull Shark *Carcharhinus leucas*	48 (32)	849.6 ± 230.1 (598–1829)	1.5 ± 0.6 (0.6–3.4)	27.9 ± 2.6 (19.7–32.9)	19.6 ± 4.0 (6.0–27.6)	6.8 ± 1.1 (4.0–9.5)	1332.2 ± 1372.8 (0.0–5698.1)	36.6 ± 6.3 (11.1–48.2)
Finetooth Shark *Carcharhinus isodon*	9	671.0 ± 288.3 (493–1435)	2.0 ± 0.2 (1.5–2.4)	26.8 ± 1.2 (26.3–29.9)	26.6 ± 1.7 (25.9–31.1)	6.4 ± 0.3 (6.0–7.1)	40.8 ± 122.4 (0.0–367.2)	8.3 ± 2.3 (3.4–12.7)
Unknown/Misidentified *Carcharhinus* sp.	44	457.4 ± 64.0 (320–582)	2.2 ± 0.5 (0.8–2.4)	26.8 ± 1.1 (22.8–28.6)	27.4 ± 3.0 (20.8–29.8)	7.4 ± 0.5 (6.5–8.9)	260.8 ± 175.9 (0.0–1048.9)	11.3 ± 9.3 (6.1–40.6)
Sand Tiger Shark *Carcharias taurus*	2 (2)	1003.5 ± 157.7 (829–1115)	3.1 ± 1.3 (2.1–4.0)	20.3 ± 7.7 (14.8–25.8)	21.4 ± 2.6 (19.5–23.2)	7.6 ± 2.4 (5.9–9.3)	1378.4 ± 1797.6 (107.3–2649.4)	21.2 ± 21.4 (6.1–36.3)
*Sandbar Shark *Carcharhinus plumbeus*	49 (33)	875.4 ± 269.1 (597–2033)	2.2 ± 0.8 (0.6–3.7)	21.8 ± 4.5 (12.3–27.5)	25.9 ± 3.5 (18.0–32.3)	7.8 ± 1.2 (5.3–11.6)	491.0 ± 679.7 (0.0–2925.0)	12.3 ± 6.7 (2.7–32.3)
Scalloped Hammerhead *Sphyrna lewini*	1 (1)	1189	2.1	27	25.3	7.9	273.5	4.4
*Smooth Dogfish *Mustelus canis*	1582 (216)	674.2 ± 112.3 (332–1228)	1.6 ± 0.6 (0.5–4.3)	25.4 ± 4.4 (10.6–32.4)	27.5 ± 2.7 (16.8–34.7)	7.7 ± 1.6 (2.0–15.6)	90.7 ± 179.4 (0.0–1880.3)	14.4 ± 8.6 (1.3–35.5)
Spinner Shark *Carcharhinus brevipinna*	3	504.0 ± 16.7 (489–552)	2.4 ± 0.6 (1.8–3.0)	28.8 ± 1.3 (27.5–30.1)	29.7 ± 0.7 (28.4–29.8)	5.2 ± 0.8 (4.6–6.1)	1131.4 ± 1079.3 (477.5–2377.2)	9.0 ± 0.6 (8.5–9.8)
*Spiny Dogfish *Squalus acanthias*	499 (99)	855.6 ± 70.4 (511–1010)	1.8 ± 0.5 (0.5–2.9)	12.9 ± 3.1 (3.4–29.9)	21.2 ± 4.8 (6.7–34.7)	9.9 ± 1.8 (6.0–14.3)	61.6 ± 124.1 (0.0–1336.0)	13.4 ± 9.1 (2.8–34.1)

**Fig 2:**
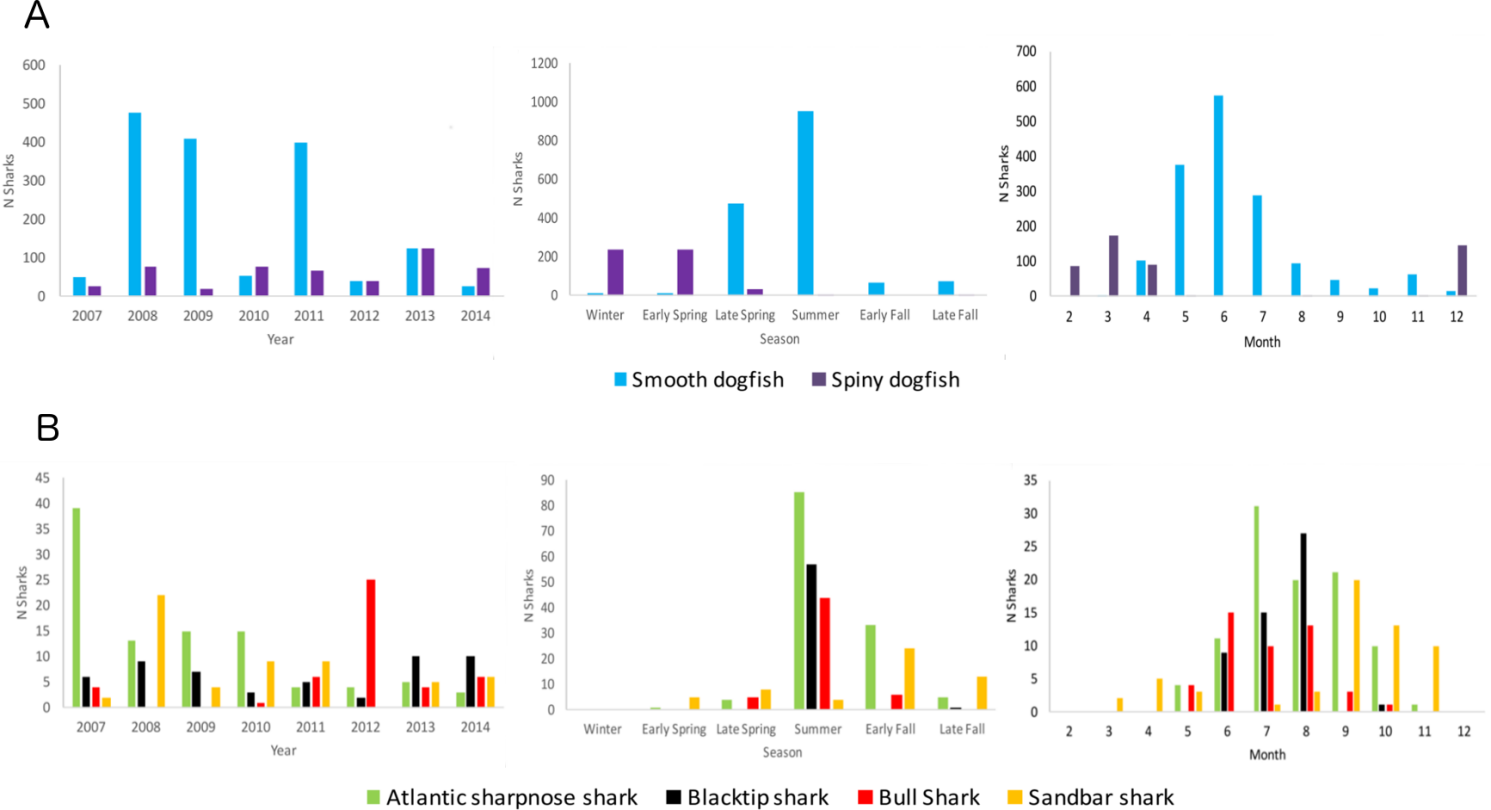
Total numbers of sharks of each principal species captured in Pamlico Sound by year, season, and month. A.) Smooth and Spiny Dogfish and B.) Atlantic Sharpnose, Blacktip, Bull, and Sandbar Sharks captured in Pamlico Sound from 2007–2014 survey years. Smooth and Spiny Dogfish are graphed separately due to being captured in much greater numbers than the other species.

Total catch was highest overall for the Smooth Dogfish, which was also the only species recorded during all seasons (Fig 2). The Spiny Dogfish was captured in greatest numbers during winter and early spring months, while Smooth Dogfish abundance peaked in early spring and summer. All other shark species were absent during the winter and most abundant during the summer with the exception of the Sandbar Shark, which was captured most often during early and late fall. Catches of Smooth Dogfish and Atlantic Sharpnose Sharks were higher during the first half of the survey period, while the majority of Bull Sharks were captured after 2011 (Fig 2). The majority of Blacktip and Sandbar Sharks fell within the immature total length range (age-1 to size at 50% maturity), more than half of the Atlantic Sharpnose Sharks were within young-of-year length range, and nearly all Spiny Dogfish were greater than the length at maturity (S1 Fig). Slightly more than 3% of Smooth Dogfish were mature, while the remainder were nearly evenly split between the young-of-year and immature size ranges (S1 Fig).

**Fig 3:**
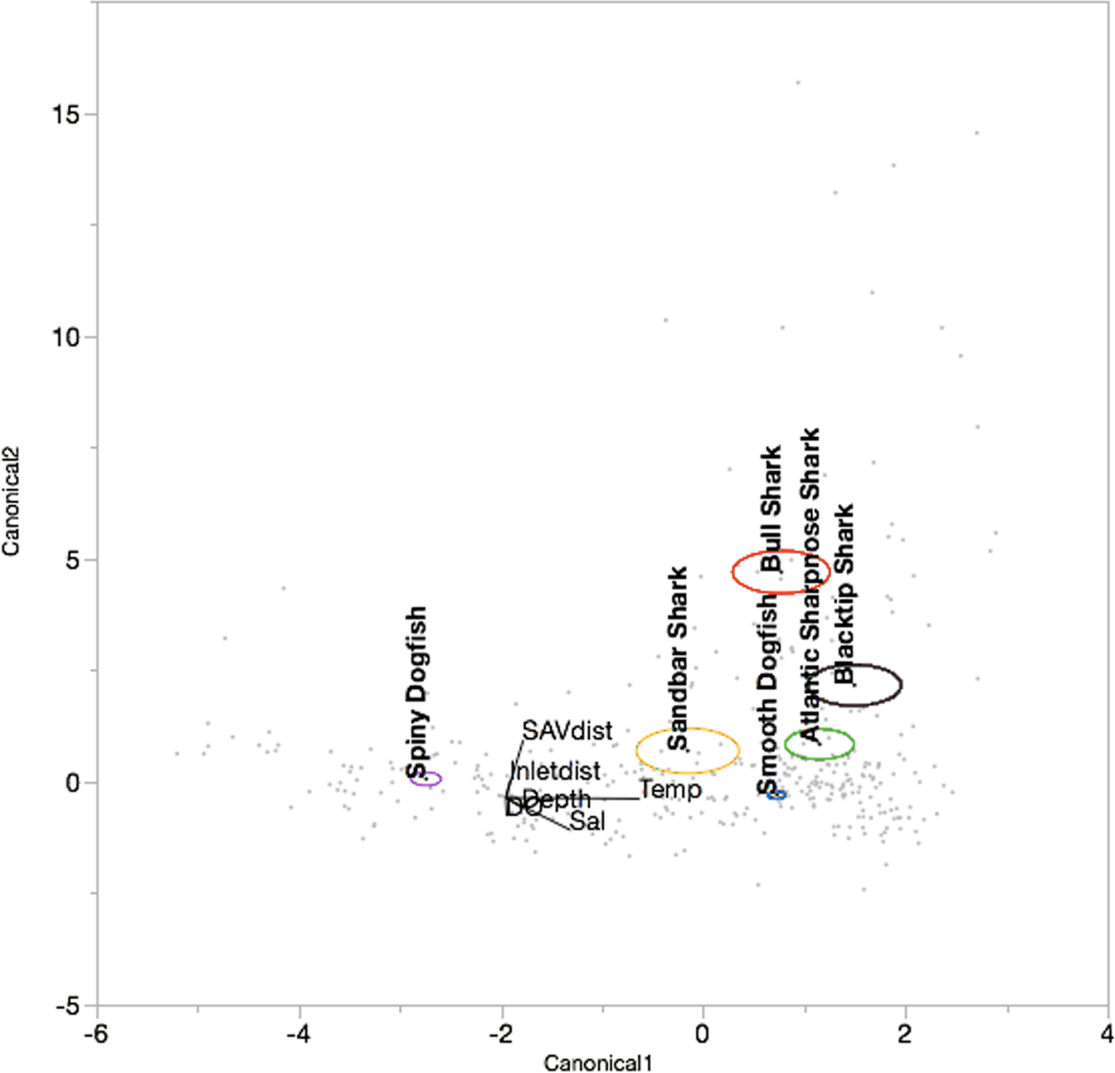
Linear discriminant function analysis canonical correspondence plot showing classification of principal shark species by environmental factors. Correlations between canonicals and environmental factors are shown in the biplot rays and ellipses represent 95% confidence intervals. Ellipses are color-coded by species (Atlantic Sharpnose Shark (green), Blacktip Shark (black), Bull Shark (Red), Sandbar Shark (gold), Smooth Dogfish (blue), Spiny Dogfish (purple)).

Linear DFA results showed 80.53% correct classification for the six principal shark species based on environmental factors (Wilks’ Lambda = 0.163, *F* = 74.87, df = 66, *p* < 0.0001). Canonicals 1 and 2 explained 94.1% of the variation (Table 2). Canonical 1 showed strong positive correlations with temperature and salinity and a strong negative correlation with dissolved oxygen, while canonical 2 showed a strong positive correlation with SAV distance (Table 2). The canonical plot showed an obvious preference for lower temperatures among Spiny Dogfish and greater SAV distance among Bull Sharks, while the other species appeared to cluster together (Fig 3). Spiny Dogfish showed the highest percentage of correct classification (92.2%), followed by Bull Sharks (91.7%) and Smooth Dogfish (82.3%) (Table 3). Exactly half of the Blacktip Sharks were correctly classified, while Atlantic Sharpnose Sharks and Sandbar Sharks were incorrectly classified as Smooth Dogfish more often than they were correctly classified (Table 3).

**Table 2:** Canonical eigenvalues, cumulative percent of variation explained, and canonical structure from linear discriminant function analysis classifying principal shark species by environmental factors.

					Canonical Structure			
Canonical	Eigenvalue	Cumulative %	Depth	Temp	Sal	DO	SAV Dist	Inlet Dist
1	2.11	71.5	-0.13	0.96	0.67	-0.64	0.18	0.04
2	0.67	94.1	0.1	0.06	-0.43	-0.11	0.86	0.34
3	0.15	99.4	0.35	-0.1	0.15	0.01	0.38	-0.9
4	0.01	99.8	-0.47	-0.18	0.52	-0.15	0.18	-0.01
5	0.01	100	0.79	-0.18	0.27	-0.25	0.13	0.23

**Table 3:** Percent of individuals of principal shark species classified as each species based on environmental factors by linear discriminant function analysis. Correct classifications are in bold.

Species			Predicted			
Actual	Atl Sharpnose Shark	Blacktip Shark	Bull Shark	Sandbar Shark	Smooth Dogfish	Spiny Dogfish
Atl Sharpnose Shark	**28.4**	16.8	1.1	4.2	45.3	4.2
Blacktip Shark	7.7	**50.0**	9.6	0.0	32.7	0.0
Bull Shark	0.0	2.1	**91.7**	2.1	2.1	2.1
Sandbar Shark	4.7	25.6	2.3	**18.6**	27.9	20.9
Smooth Dogfish	0.3	1.2	0.0	9.8	**82.3**	6.4
Spiny Dogfish	0.2	0.0	0.0	4.4	3.2	**92.2**

### Environmental data processing

**Fig 4:**
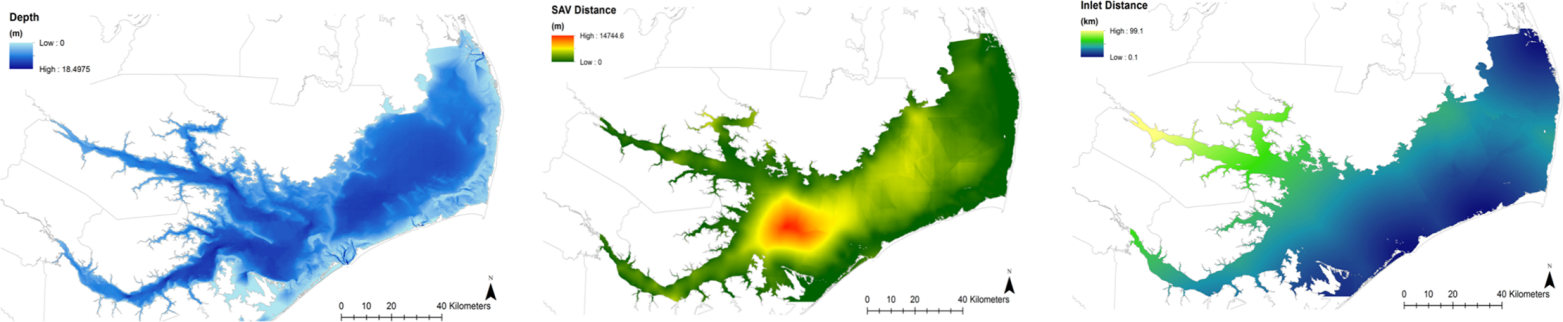
Environmental grids for Pamlico Sound representing habitat factors assumed to be static. Depth data are from USGS bathymetry surveys and inlet distance and SAV distance are interpolated from 2007–2014 NCDMF fishery-independent surveys and spatial locations of inlets and SAV habitat. SAV habitat locations are from Kenworthy et al. [34].

**Fig 5:**
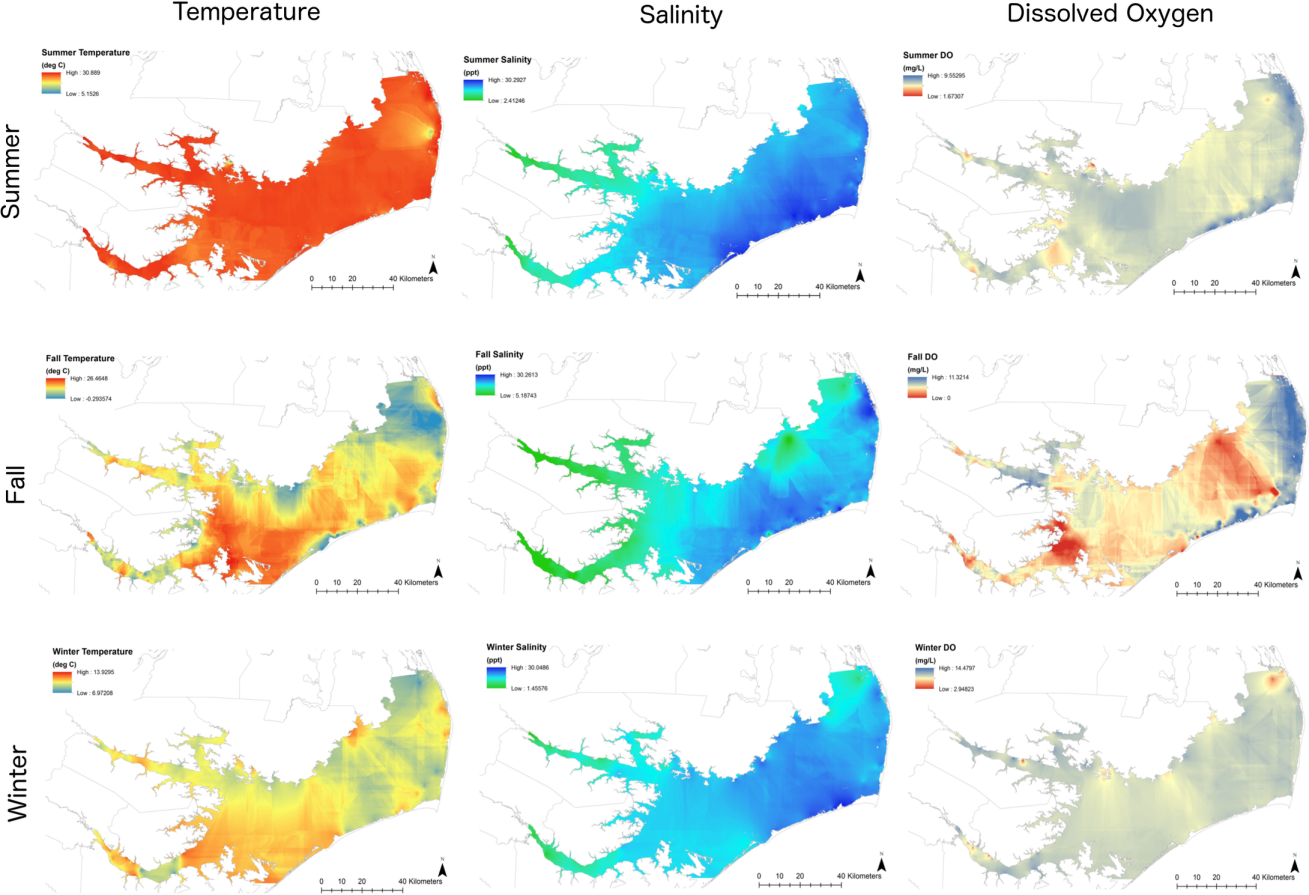
Environmental grids for Pamlico Sound representing environmental factors assumed to vary by season. Seasons are those in which the greatest seasonal abundance of at least one principal shark species was recorded and fall represents early and late fall combined. Data are interpolated from North Carolina Division of Marine Fisheries 2007–2014 fishery-independent surveys.

Multiple linear correlation analyses showed that all correlations between environmental factors were statistically significant (*p* < 0.05). Strong negative correlations (*r* < -0.5) were found between temperature and dissolved oxygen and between inlet distance and salinity (Table 4). Depth, SAV distance, and inlet distance were assumed to be static over the survey period and grids of these habitat factors were not specific to season (Fig 4). Temperature, salinity, and dissolved oxygen measurements recorded during summer, fall (early and late fall combined), and winter were used to create environmental grids within Pamlico Sound (Fig 5). Environmental grids showed differences in the spatial distribution of temperature, salinity, and dissolved oxygen measurements, though warmer temperatures, higher salinity, and higher dissolved oxygen were consistently found in the southern and eastern portions of Pamlico Sound (Fig 5). Unrepresentativeness was relatively low and distributed mostly evenly across the sound during the summer but appeared to follow depth contours during the fall and winter, during which higher unrepresentativeness occurred in the eastern and northern portions of the estuary (S2 Fig).

**Table 4:** Linear correlations between environmental factors recorded during 2007–2014 North Carolina Division of Marine Fisheries gillnet and longline surveys in Pamlico Sound.

	Depth	Temp	Sal	DO	SAV Dist	Inlet Dist
Depth	1.00					
Temp	0.11	1.00				
Sal	0.15	0.07	1.00			
DO	-0.13	-0.54	-0.03	1.00		
SAV Dist	0.24	0.10	-0.04	-0.10	1.00	
Inlet Dist	-0.14	-0.04	-0.67	-0.03	0.06	1.00

### BRT modeling

Environmental variables contributing the most tree splits to BRT models differed between species (Table 5). Temperature was among the top three contributors to the binary model for all principal species and inlet distance was among the top contributors for all species except the Sandbar Shark. Though temperature was a major contributor of tree splits for all species, the Spiny Dogfish was the only species for which it was the top contributor. A greater percentage of tree splits for the Sandbar Shark were based on dissolved oxygen and salinity than temperature. Dissolved oxygen also contributed a greater percentage of tree splits than temperature for the Atlantic Sharpnose Shark. For the Blacktip Shark, SAV distance accounted for a percentage of tree splits nearly equal to that of inlet distance and temperature, while this variable was the lowest contributor of the top three environmental variables for the Bull Shark. Salinity was the greatest contributor for the Smooth Dogfish, and despite not being included within the top three contributors in the final model, SAV distance replaced temperature within the top three variables in a third of the model runs. Depth contributed the lowest percentage of tree splits for all species (Table 5).

**Table 5:** Parameters (cross-validation score (CV), learning rate (lf), bag fraction(bf)) and contribution (% of tree splits) for binary BRT models predicting capture likelihood of six principal shark species in Pamlico Sound. Bold text denotes the three environmental factors contributing the most tree splits for each species.

		Binary Model					% Contribution			
Species	CV	lr	bf	N Trees	Depth	Temp	Sal	DO	SAV Dist	Inlet Dist
Atl Sharpnose Shark	0.709	0.001	0.6	2250	4.25	**20.03**	14.51	**21.89**	16.07	**23.25**
Blacktip Shark	0.826	0.00075	0.6	3750	2.48	**22.21**	16.77	10.45	**24.11**	**23.97**
Bull Shark	0.722	0.001	0.5	1850	3.82	**19.28**	14.65	14.98	**16.05**	**31.23**
Sandbar Shark	0.791	0.001	0.6	2900	9.61	**17.58**	**18.46**	**26.36**	10.71	17.26
Smooth Dogfish	0.771	0.0075	0.6	1350	4.38	**17.34**	**33.45**	9.53	16.32	**18.97**
Spiny Dogfish	0.771	0.001	0.5	5950	8.41	**29.60**	**13.40**	11.28	11.70	**25.60**

**Fig 6:**
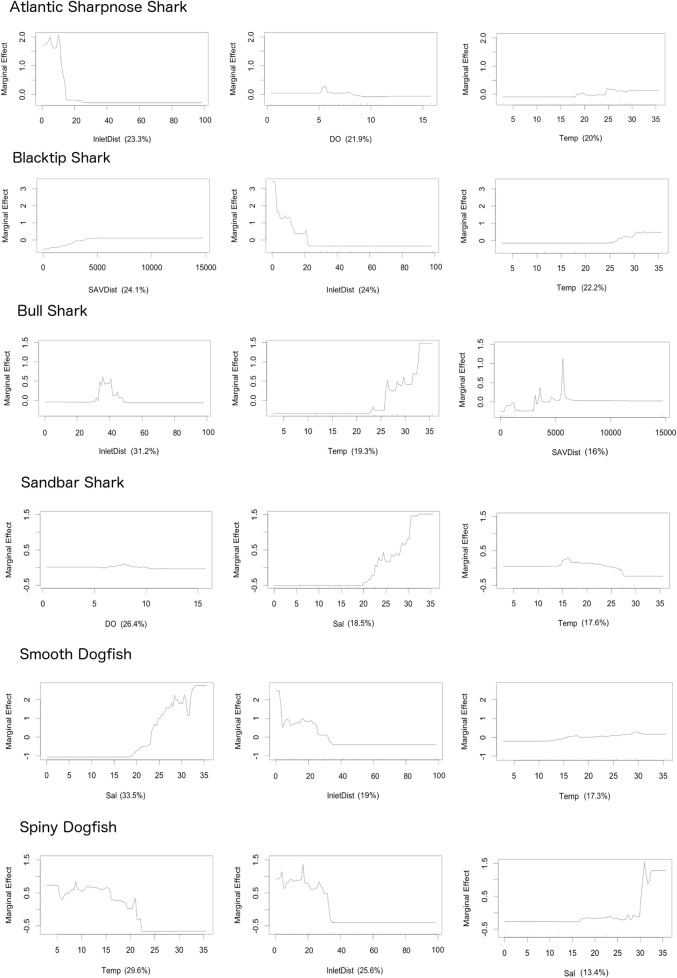
Marginal effects of environmental factors contributing to predicting shark species capture likelihood. Marginal effects are of the three environmental factors that contributed the three highest proportions of tree splits to the binary BRT model predicting capture likelihood of each of the six principal shark species within Pamlico Sound. Percentages in parentheses represent the proportion of tree splits contributed by the variable.

Marginal effect plots of binary BRT models showed consistencies and differences between species in the effect of the top three environmental factors on capture likelihood (Fig 6). A relatively short inlet distance (< 40 km) showed a positive effect on capture likelihood for all species except the Bull Shark. Temperatures greater than 15°C were positively associated with capture likelihood for all species except the Spiny Dogfish, though temperatures greater than 27°C showed a subsequent negative association with Sandbar Shark presence. For species with salinity as one of the three environmental factors contributing most to the binary model, salinities greater than 20 ppt had a positive effect on capture likelihood (Fig 6).

**Fig 7:**
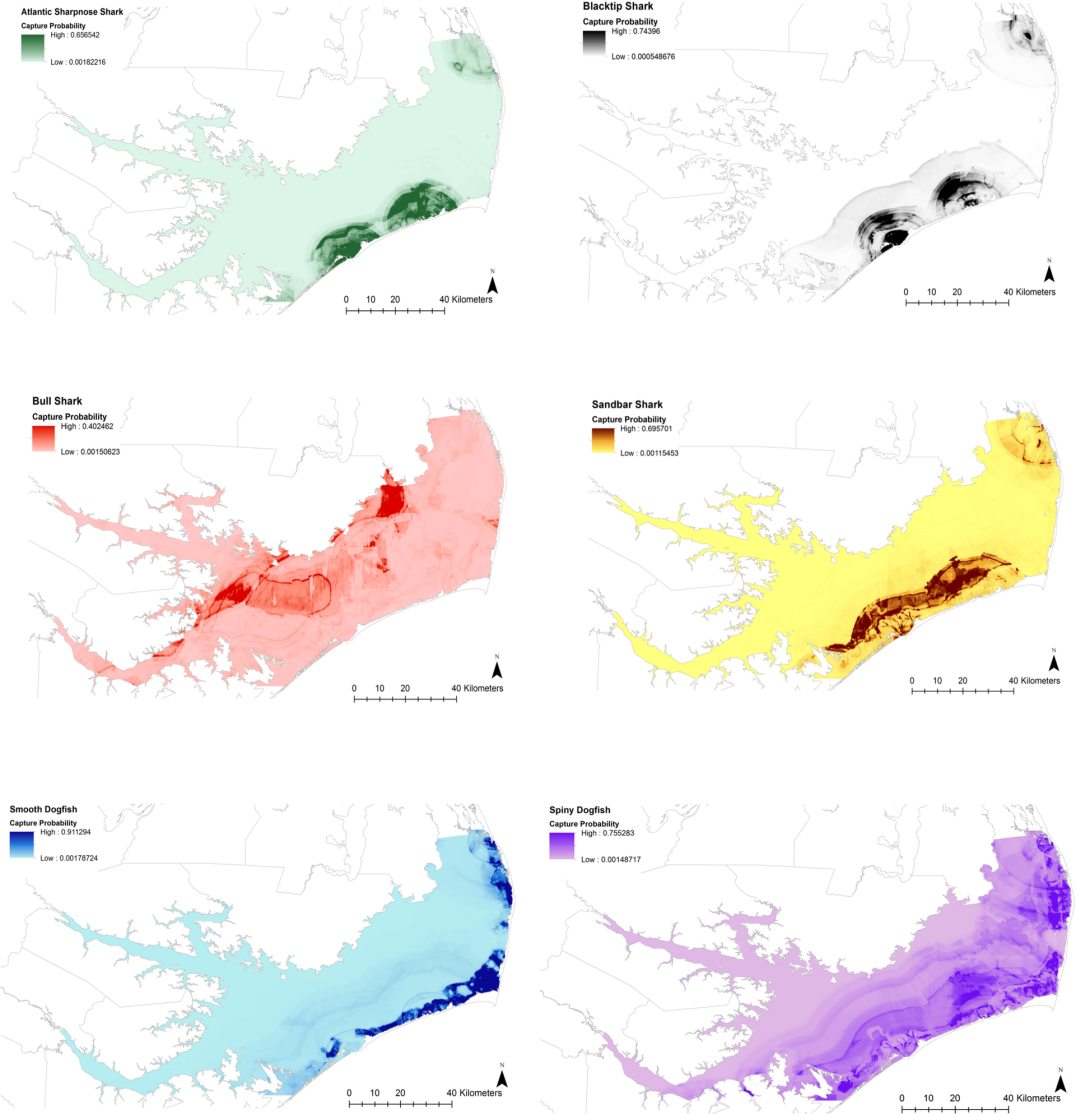
Predicted capture likelihood maps for the six principal shark species within Pamlico Sound. Predicted capture likelihood is based on binary presence/absence BRT results.

Predicted habitat maps explicitly showed the importance of inlet distance, with four of the six principal shark species predicted to occur within a relatively well-defined distance from the inlets (Fig 7). While predicted capture likelihood of Atlantic Sharpnose and Blacktip Sharks was primarily associated with the inlets, Sandbar Shark and Spiny Dogfish capture likelihood also showed associations with depth contours. The models predicted high Smooth Dogfish capture likelihood primarily along the eastern Pamlico Sound in an area coinciding with extensive seagrass beds within eastern Pamlico Sound, though SAV distance was not among the highest contributors to BRT splits. The Bull Shark was the only species with areas with high capture probabilities in the western portions of the estuary, with two distinct “hot spots” at the Long Shoal River between the Pamlico River and Stumpy Point and southeast of the mouth of the Pamlico River (Fig 7). Kruskal-Wallis test results showed that predicted capture likelihood was significantly higher at the locations of sets from 2015 NCDMF gillnet and longline surveys where each species was present, though results for Smooth Dogfish were marginally significant at an α = 0.05 significance level (Table 6).

**Table 6:** Kruskal-Wallis test results comparing predicted CPUE at the locations of sets from 2015 NCDMF gillnet and longline surveys where each species was present or absent (df = 1 for all tests).

Kruskal-Wallis test results		Predicted Capture Likelihood			
Species	N Sets Present	Present (Mean ± SD)	Absent (Mean ± SD)	*X*^*2*^	*p*
Atlantic Sharpnose Shark	12	0.051 ± 0.049	0.014 ± 0.033	19.90	<0.0001
Blacktip Shark	3	0.053 ± 0.039	0.004 ± 0.014	8.14	0.004
Bull Shark	7	0.016 ± 0.021	0.007 ± 0.010	6.58	0.010
Sandbar Shark	4	0.037 ± 0.055	0.006 ± 0.014	7.11	0.008
Smooth Dogfish	2	0.320 ± 0.023	0.079 ± 0.166	3.85	0.050
Spiny Dogfish	7	0.168 ± 0.109	0.043 ± 0.066	10.09	0.001

## Discussion

The results of this study show that Pamlico Sound provides habitats for a diverse and dynamic assemblage of shark species, with principal species known to occur in estuarine or nearshore environments in both temperate waters to the north (Sandbar Shark, Smooth Dogfish, Spiny Dogfish) and subtropical systems to the south (Atlantic Sharpnose Shark, Blacktip Shark, Bull Shark) [42]. The highest shark diversity occurred during the summer, but sharks were also present during cooler months, with Spiny Dogfish most abundant during winter and Sandbar Sharks occurring primarily in fall months. Shark presence within the estuary was primarily associated with distance from the inlet and temperature while abundance appeared to be associated with salinity, though other environmental factors were of importance on a species-specific basis. BRT models identified the inlets as important landmarks in delineating shark habitat for most of the principal species, though Bull Sharks appeared to associate with river mouths and Smooth Dogfish abundance closely overlapped seagrass beds on the estuarine side of the Outer Banks. Bull Sharks and Smooth Dogfish showed the most distinctive habitat preferences while Spiny Dogfish, Atlantic Sharpnose, Blacktip, and Sandbar Sharks showed similar associations with inlet distance and salinity but differed based on temperature preferences.

The assemblage of shark species within Pamlico Sound is made up of species known to occur in other nearshore and estuarine habitats in the Mid-Atlantic and Southeastern regions of the U.S. East Coast [8,43] and the seasonal and monthly occurrences of the principal species follow the timing of seasonal migrations. Blacktip Sharks undergo large-scale seasonal migrations, with peak abundance in North and South Carolina waters typically occurring during the summer and southeastern Florida during the winter [44,45]. Conversely, Spiny Dogfish peak capture likelihood occurred during winter, when the distribution of this species extends south of Cape Hatteras [46,47]. Perhaps the best-known example of overwintering habitat for a shark species in North Carolina is the migration of Sandbar Sharks into an area south of Cape Hatteras during winter [48–50]. However, catches of Sandbar Sharks were highest during the early and late fall but completely absent during winter, suggesting that this species may enter the sounds as part of their migration from summer habitats but may move out to nearshore oceanic waters in the peak of winter.

Based on the predominance of juvenile size classes within the Pamlico Sound shark assemblage, this estuary may serve as nursery habitat for several species, particularly the Smooth Dogfish. The size distribution of this species was strongly skewed towards neonate and early juvenile sizes [51] and peak capture probability in the late spring and summer coincided with parturition timing and nursery habitat use in other estuaries [52]. Given the high capture probability of Smooth Dogfish in NCDMF surveys alone, eastern Pamlico Sound may be worthy of consideration as essential habitat for this species. The presence of neonate Bull Sharks is noteworthy due to a lack of evidence for nursery habitat for this species within Pamlico Sound prior to 2011 [53]. Neonate Bull Sharks occurred within the time frame of parturition in Florida’s Indian River Lagoon, a confirmed primary nursery [54], suggesting that these individuals were born within the estuary and did not migrate from elsewhere. Neonate Sandbar Sharks are likely migrants from primary nurseries in the Chesapeake and Delaware Bays [49,55] but there is evidence for limited use of North Carolina waters as a resident primary nursery [56].

Discriminant function analysis showed there was little habitat overlap between the Bull Shark or Spiny Dogfish and other shark species in Pamlico Sound. The main drivers of these habitat differences appeared to be water temperature for the Spiny Dogfish and inlet distance or salinity for the Bull Shark. These results are in keeping with preferences for low temperatures among Spiny Dogfish [36,47] and for comparatively low salinities and greater inlet distances among Bull Sharks [37,57,58] documented in other systems. The majority of misclassifications were other species classified at Smooth Dogfish, likely a result of the abundance of this species within the survey catches and its presence during all seasons. Though Smooth Dogfish were the third most correctly-classified species, this may be a function of the large sample size reducing uncertainty for this species, as it was classified within the same canonical biplot space as the species with lower correct classifications. The low classification success among the remaining species may be an indication of similar or overlapping habitat preferences among Atlantic Sharpnose, Blacktip, and Sandbar Sharks in Pamlico Sound. High misclassification rates between particular species may also represent seasonal overlap. For example, there was high misclassification between Atlantic Sharpnose and Blacktip Sharks, two species that were most abundant during summer months. The Sandbar Shark was less associated with warm temperatures than the other Carcharhinid species and showed nearly equal misclassification as Blacktip Sharks or Spiny Dogfish, which were almost exclusively warm and cold temperature species, respectively. This may reflect a broad temperature range for the Sandbar Shark, leading to a broader seasonal presence and the potential for habitat overlap with species in both the warm and cold-water shark assemblages.

BRT model results and the resulting predicted capture probability maps identified the most important environmental factors structuring the Pamlico Sound shark assemblage and confirmed many of the habitat associations found during DFA. Inlet distance and temperature were strongly associated with presence for the majority of species, suggesting that these may be the main drivers of shark presence within Pamlico Sound. Given the strong correlation between inlet distance and salinity, it is likely that inlet distance preferences are directly related to salinity preferences, and the influence of these two factors may be interchangeable. However, inlet distance preferences may also be influenced by biotic factors such as prey availability or avoidance of predators or competitors that were not directly measured. Other studies of estuarine shark habitat use have found that inlet distance can serve as a proxy for salinity preference when delineating habitat and may actually be more useful from a management perspective [33,37]. Both temperature and salinity are major influences on shark habitat selection: temperature-associated movements are widespread among shark species and the majority of sharks tolerate a relatively narrow range of high salinities [59]. Broadly, seasonal shark presence within Pamlico Sound is likely driven by temperature while the spatial extent of habitat available within the estuary is probably defined by salinity. However, the influence of other environmental factors may be important in defining habitat on a more local and species-specific basis.

The effects of temperature and salinity on predicted capture probability matched the unique habitat preferences for Spiny Dogfish and Bull Sharks identified by linear DFA. Increasing temperature had a positive effect on presence for all species except the Spiny Dogfish, while increasing salinity or decreasing inlet distance had a positive effect on the presence of all species except the Bull Shark. The ability of Bull Sharks to utilize a broader salinity range than other species has been noted in several studies of multispecies shark assemblages [37,58,60]. This adaptation may allow juvenile Bull Sharks to occupy portions of estuarine nursery areas that are inaccessible to other shark species, allowing them to reduce competition and predation risk [11]. Effects of temperature and salinity on Spiny Dogfish capture probability are interesting. Temperature preferences reflect seasonal presence, as Spiny Dogfish migrate into North Carolina waters during the winter [46] when most other species are absent from Pamlico Sound. However, other shark species are known to occur within the coastal oceanic waters just outside the estuary during the winter, including large numbers of juvenile Sandbar and Dusky (*Carcharhinus obscurus*) Sharks [48]. The increased capture probability of Spiny Dogfish at mid-range salinities suggests that this species enters the estuary to avoid competition with other sharks, though observations from local fishermen also suggest that Pamlico Sound may represent a cool water refuge during periods when the Gulf Stream is in close proximity to shore (Chris Hickman, F/V Bout Time, pers. comm.).

Capture probability maps may have also identified important geographic features for the Bull Shark and Smooth Dogfish. Bull Shark capture probability was greatest at two potential habitat hotspots in relatively close proximity to sources of freshwater inflow: one at the Long Shoal River approximately equidistant from the mouth of the Pamlico River and the entrance to the Albemarle Sound, and one east of Goose Creek Island between the mouths of the Pamlico and Neuse Rivers. As mentioned previously, lower-salinity habitats may provide a low-mortality environment for juvenile Bull Sharks by reducing the likelihood of interacting with other sharks [11]. Also, habitat near freshwater outflows may allow the juvenile Bull Sharks access to pulses of prey from freshwater sources [61]. The majority of potential Smooth Dogfish habitat occurred within areas of extensive seagrass coverage along the sound side of the Outer Banks. Though juvenile Smooth Dogfish are unlikely to be small enough to take shelter within seagrass, these habitats may provide increased foraging opportunities. Smooth Dogfish primarily feed upon crustaceans such as crabs and shrimp [52,62], and many crustacean species make extensive use of seagrass habitat [3,63]. Other mobile crustacean predators such as Red Drum (*Sciaenops ocellatus*) and Bonnethead Sharks (*Sphyrna tiburo*) are known to associate with seagrass beds [64,65]. Therefore, the extensive seagrass meadows in the eastern portions of Pamlico Sound may be critical foraging habitat for juvenile Smooth Dogfish, and populations of this species may be impacted by degradation or restoration of seagrass beds. However, habitat preferences based on foraging opportunities cannot be confirmed without data on prey distributions. Further, habitat selection may not match prey distributions if predator avoidance is prioritized over foraging opportunities [14], or if environmental preferences limit overlap between shark and prey distributions.

The importance of inlet distance for four of the six principal shark species was obvious. Capture probability of the Atlantic Sharpnose Shark, Blacktip Shark, Sandbar Shark, and Spiny Dogfish peaked within a certain radius of Oregon, Hatteras, and Ocracoke Inlets, but was otherwise low elsewhere in Pamlico Sound. These species are primarily coastal sharks associated with nearshore or lower estuarine environments [36,47,60,66] and are likely transient within Pamlico Sound, making short foraging trips into the estuary from the ocean. Blacktip Sharks in Texas estuaries were predicted to occur exclusively near tidal inlets [37] and a study of shark habitat in the Florida panhandle that included both estuarine and nearshore areas predicted the highest rates of occurrence for Atlantic Sharpnose and Blacktip Sharks at nearshore areas open to the Gulf of Mexico [67]. Potential juvenile Sandbar Shark habitat in the Chesapeake Bay was found to be limited to within 34.5 km of the estuary mouth [33]. Though salinity preferences prevent these shark species from penetrating far into the estuary, the extremely limited access through the inlets creates geographic bottlenecks that may facilitate important ecological interactions between these sharks and any migratory prey or predator species transiting between the ocean and Pamlico Sound. Similar circumstances may increase predation risk for species leaving estuaries after spawning or reaching their marine life stage, and several shark species are known to occur at estuary entrances during periods of mass migration out of the estuary [68,69].

This study is one of relatively few to define and predict elasmobranch habitat using environmental and spatial factors. An early example by Grubbs and Musick [33] used regression tree analysis to spatially delineate juvenile Sandbar Shark habitat within the lower Chesapeake Bay. Froeschke et al. [37] followed this example by using BRT analysis to create predicted abundance models for three shark species in Texas estuaries, then Dedman et al. [38] developed the *gbm*.*auto* script for R to automate the BRT analysis and mapping processes while identifying habitat for species in the Irish Sea skate complex. Other modeling approaches used to predict spatial habitat extent for elasmobranchs include the use of generalized additive models to identify Spiny Dogfish habitat in the northwest Atlantic [70] and generalized linear mixed models to spatially delineate habitat for six shark species in the northeastern Gulf of Mexico [67].

The BRT approach was most appropriate for the data used in this study due to its flexibility and statistical power in situations with zero-inflated distributions and relatively low sample sizes [39]. Despite this, there were some limitations to our analysis. The lack of survey sets beyond the 2-m depth contour lead to undersampling of much of the interior, deep-water area of the estuary. However, unrepresentativeness maps showed approximately equal representation across the entire sound, with the greatest unrepresentativeness occurring during winter in areas that were covered by the longline survey during other seasons. Identification of potential habitat areas is one of the main purposes of BRT modeling. While it is possible the habitat models generated during this study may not reflect the full influence of depth or environmental associations found in the deep areas of Pamlico Sound, unrepresentativeness seemed to be associated more with well-sampled but highly variable areas near the inlets than the unsampled areas in the center of the estuary. Finally, while interannual variation was obvious for some species, low annual sample sizes for most species precluded detailed analysis of the effect of year on capture probability.

## Conclusion

Shark species presence and community composition within Pamlico Sound appears to be largely influenced by temperature, while spatial habitat extent is a function of salinity in combination with other environmental factors. Both temperature and salinity can vary significantly over space and time under the influence of weather or climatic conditions, so the location and extent of shark habitat may shift in response to rising water temperatures and increased precipitation associated with climate change [71]. Large-scale intraspecific and interspecific associations between coastal sharks have also been observed in the northwest Atlantic and may influence the timing and extent of species distributions within the estuary [72]. Because of its enclosed and seasonally dynamic nature, Pamlico Sound has great value as a system where hypotheses about the effects of climate shifts and the relative importance of abiotic and biotic factors can be tested.

Sharks are clearly a part of the Pamlico Sound estuarine community and patterns of habitat use within the estuary may reflect or facilitate interactions with other species, including economically important teleost and crustacean species. Pamlico Sound shares many characteristics with other systems in which sharks have been identified as an important influence on community dynamics and population structure for other species [16,68,69]. With the identification of areas regularly used by sharks, further work should assess potential habitat overlap with other species and the nature and extent of ecological interactions involving sharks in Pamlico Sound and determine if those interactions generalize across estuaries.

## Supporting information

S1 Fig Proportions of sharks of each principle species within each life stage. Proportions are of the total number of sharks captured from both NCDMF gillnet and longline surveys combined. Life stages were classified based on length-at-age estimates from age and growth studies from each species.
(TIF)

S2 Fig Maps of environmental unrepresentativeness for each season. Unrepresentativeness measures representation of the full range of data from environmental rasters generated for summer, fall, and winter. Seasons were chosen due to being the season of peak abundance for at least one principle coastal shark species.
(TIF)
