# Supplementary figures and images for: Delineation and mapping of coastal shark habitat within a shallow lagoonal estuary
Source: PLoS One. 2018 Apr 12;13(4):e0195221. doi: 10.1371/journal.pone.0195221 (PMC5896943; doi:10.1371/journal.pone.0195221)

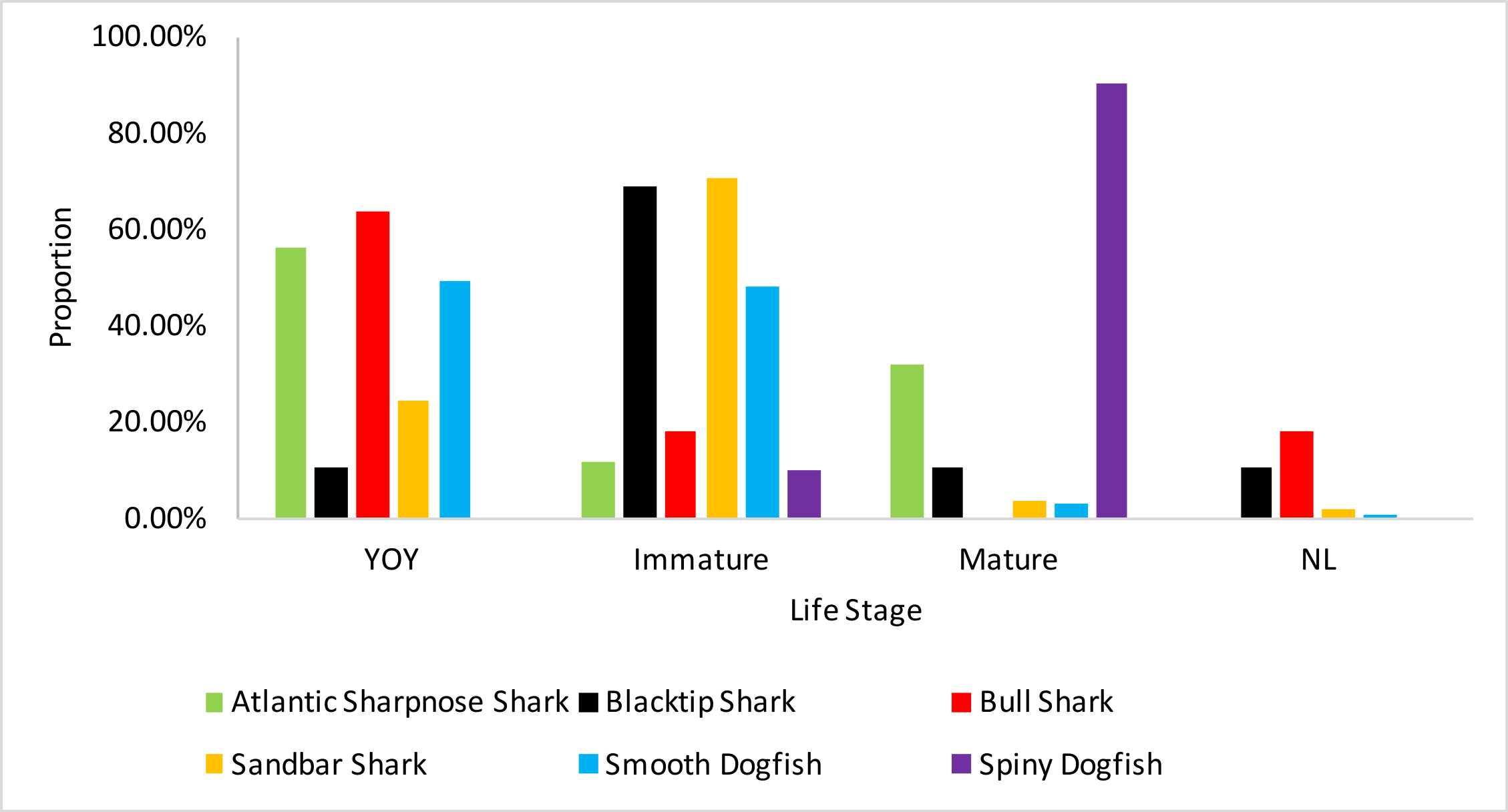

Supplement: S1 Fig — Proportions are of the total number of sharks captured from both NCDMF gillnet and longline surveys combined. Life stages were classified based on length-at-age estimates from age and growth studies from each species. (TIF) [file pone.0195221.s001.tif]

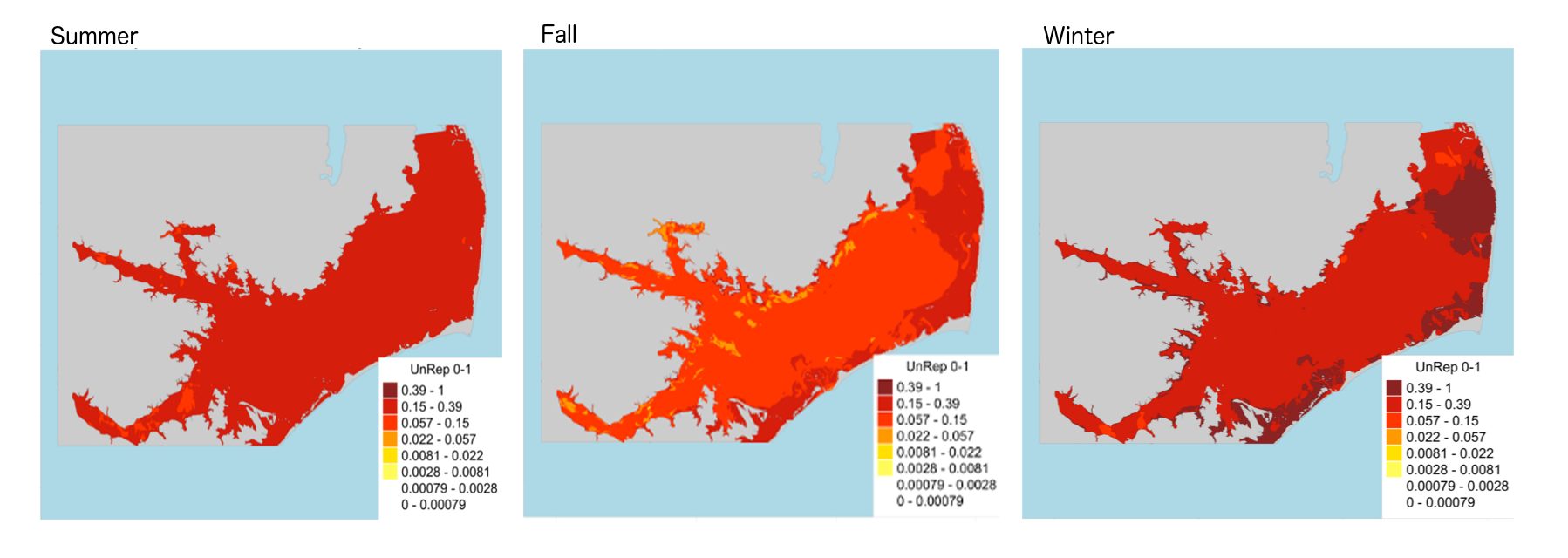

Supplement: S2 Fig — Unrepresentativeness measures representation of the full range of data from environmental rasters generated for summer, fall, and winter. Seasons were chosen due to being the season of peak abundance for at least one principle coastal shark species. (TIF) [file pone.0195221.s002.tif]
